# Factors Influencing Users’ Perceived Restoration While Using Treetop Trails: The Case of the Fu and Jinjishan Forest Trails, Fuzhou, China

**DOI:** 10.3390/ijerph19042242

**Published:** 2022-02-16

**Authors:** Yuanjing Wu, Qianyun Li, Hanqing Zheng, Shijie Luo, Qunyue Liu, Zhao Yan, Qitang Huang

**Affiliations:** 1 College of Landscape Architecture, Fujian Agriculture and Forestry University, Fuzhou 350100, China; fjwuyuanjing@126.com (Y.W.); li579ll@126.com (Q.L.); fafuzhq@126.com (H.Z.); hakcanlsj@163.com (S.L.); 2 College of Architecture and Urban Planning, Fujian University of Technology, Fuzhou 350118, China; fafulqy@outlook.com; 3 Xiamen Shangyimeihui Education Consulting Co., Ltd., 709, Siming District, Xiamen 361000, China; fjyanzhao@126.com

**Keywords:** treetop trails, greenways, perceived restoration, place attachment, environmental quality

## Abstract

Studies have indicated that urban greenways promote physical and perceived restoration. However, there is a lack of research on the impact of treetop trails on human perceived restoration. In this study, two representative treetop trails in Fuzhou city were selected to investigate treetop trails’ impact on users’ perceived restoration. The study adopted a structural equation modelling approach to explore the influence mechanisms and pathways of treetop trails on users’ perceived restoration, through 412 questionnaires. The results showed that the perceived environmental quality of treetop trails had a significant positive effect on users’ overall psychological wellbeing. Place attachment had a significant positive effect on users’ perceived restoration and a significant mediating effect on users’ perceived environmental quality of trails. The results of this study revealed that the mechanisms of the impact of treetop trails on users’ perceived restoration and the construction of treetop trails can be enhanced in the future by improving trail facilities, enriching trail perception of elevated feeling, improving trail landscape quality, and optimising trail design.

## 1. Introduction

In modern society, people face a range of challenges that may lead to psychological and other health issues [1]. Numerous studies have demonstrated that urban green space can promote health [2,3,4]. The mechanisms of the positive relationship between green spaces and health include alleviation of mental stress, enhancement of social cohesion, promotion of physical activity, and mitigation of potentially hazardous environmental exposures (such as air and noise pollution) [5]. Urban greenspace includes different components, such as parks, gardens, greenways, forests, wetlands, green roofs, and green walls [6]. Among them, urban greenways, as linear green spaces established either along a natural corridor or overland along a railroad right-of-way [7], have received increasing attention. In particular, a popular elevated steel pedestrian walkway system weaving through forested areas in Fuzhou, China, which provides people with access to the city’s mountain and forest areas [8], signifies an awakened consciousness and desire to pay attention to urban greenways [8].

Urban greenways are public resources that enhance the multifaceted functionality of urban green space, such as improving biodiversity, providing recreation areas, and conserving cultural heritage [7]. Numerous studies have illustrated the significant role of urban greenways in facilitating human health [9]. Greenways can provide urban residents with opportunities for interacting with nature, physical activity, and stress reduction [10,11], thereby promoting their health and wellbeing [12,13]. Involvement in sports and exercise not only provides individuals with health benefits but has positive effects on mood [14]. Although previous studies have demonstrated the health benefits of urban greenways, only a few have investigated the health impacts of forest trails in China. Research conducted by Huang et al. in the Fu Forest Trail in Fuzhou (usually known as Fudao) revealed that participants’ restorative experience in the Fu Forest Trail positively contributed to perceived health [15]. In addition, previous studies have concluded that greenway location, length, width, pavement, facilities, accessibility, and surrounding features environmental factors affect the use of urban greenway [16,17,18]. In terms of the forest trails, recreational involvement has been identified as a key factor that affects participants’ wellbeing [19]. Their study further revealed that communing with nature, relaxing the body and mind, exploring novelty, keeping health, entertaining, developing personal interest, making communication, and seeking knowledge were the main purposes of the participants for visiting forest trails [20]. However, few have discussed the impact of the environmental quality of forest trails on users’ perceived health. Moreover, studies concerning urban greenways in China have mainly focused on introducing the planning and construction of Western greenways, the ecological benefits of greenways, and the patterns and factors of urban greenway use [21], while attaching less importance to public perception. In particular, the process of place attachment, which influences people’s emotions, meanings, and behaviours has been largely ignored [22].

Furthermore, studies have found that the environmental quality of urban green space impacts users’ place dependence and place identity, which further impacts visitors’ perceived restoration with urban green space [23,24]. However, we do not know whether this kind of relationship can also be applicable to forest trails. Therefore, this study investigated forest trails in Fuzhou to examine the effects of environmental quality on users’ place attachment, and perceived restoration. This study sought to determine:

**O1.** The effect of greenway quality on users’ perceived restoration;
**O2.** The effect of greenway quality on users’ place attachment;
**O3.** The effect of place attachment on users’ perceived restoration; and
**O4.** The role of place attachment in the relationship between greenway quality and perceived restoration.

The results of this study can examine the positive outcomes of a new kind of urban greenway and could be conducive to the optimisation and promotion of treetop trails in the future.

## 2. Theoretical Background and Hypothesis

### 2.1. Greenway Quality in Relation to Perceived Restoration

Greenways have evolved in response to the physical, cultural, political, and psychological pressures of urbanisation [25,26]. The term greenway takes on different names in different parts of the world. For example, they are known as ‘green corridors’ in European countries and are referred to as ‘corridors verdes’ in Portugal [25]. Regardless of their many diverse forms or terms across the world and their design and structure, greenways represent “multiple objective, open space corridors that perform natural functions while offering desirable aesthetic qualities to humans as they recreate or commute along trails” [27]. Due to their linear nature, urban greenways can facilitate exercise such as walking, jogging, and cycling, and create opportunities for positive interactions between humans and nature in congested cities [28]. The main factors that affect urban greenways use are individual and environmental factors and use patterns [21]. In addition, trail paths, rest benches, restrooms, and other service facilities can increase the attractiveness of an urban green space, thus increasing the time that users spend there [29]. The greenway qualities within environments help promote restorativeness [27,30]. Chang et al.’s study in Taizhong City, Taiwan, indicated that the overall quality of the greenway positively contributed to users’ mental health and wellbeing [9]. Another study conducted by Liu et al. (2018) [31] revealed that the quality of green space affected users’ perceived restoration and health. Users’ perceived restoration refers to recovering from mental fatigue and the negative emotions associated with stress [32,33]. According to Kaplan and Kaplan [34], perceived restoration includes four components, namely: (a) being away: conveying a sense of escape from the source of stress and fatigue; (b) extent: presenting opportunities for physical or mental exploration; (c) fascination: boosting feelings of fascination and wonder; and (d) compatibility: being compatible with individual users’ needs and preferences. These four components provide insights into how their existence within greenways are more likely to encourage psychological restoration. Therefore, based on previous findings, this study proposes the following hypothesis:

**Hypothesis** **1** **(H1).** *Greenway quality positively contributes to users’ perceived restoration.*

### 2.2. Greenway Quality in Relation to Place Attachment

*Place attachment* refers to the emotional bond people have with a particular place [35], which has also been conceptualised as a sense of place, place bonding, or place identity. The establishment of place attachment helps individuals regulate their emotions. Many studies have depicted place attachment as place dependence and place identity [35,36,37]. Place dependence refers to a functional attachment to specific environments, reflecting a setting’s ability to provide features and conditions that meet users’ goals or desired activities [38]. With a higher level of satisfaction, individuals’ bonding with that environment will be deeper [39]. Place identity is defined as the complex emotional bonding with, and sense of belonging to, a certain place, which is regarded as an extension of oneself and a contributor to personal and social identities [40]. It reflects individuals’ attitudes, thoughts, values, beliefs, meanings, and behavioural tendencies regarding the environment [41]. Place attachment is affected by age, location, and climate [42,43,44], as well as environmental quality [42]. Although few studies explored the associations between greenway quality and place attachment, similar studies conducted in green space revealed that environments with better quality result in a stronger place attachment [9]. For example, with data collected from 348 participants across Reunion Island, Junot found that the environmental quality of Reunion Island was strongly related to neighbourhood attachment [45]. Their study further indicated that characteristics of the urban park landscape contributed to visitors’ place dependence and place identity. Moreover, the study has initially found that recreational involvement with forest trails can enhance visitors’ place attachment with the trail [19]. Therefore, based on this literature, this study proposed the following hypothesis:

**Hypothesis** **2** **(H2).** *Greenway quality positively contributes to place attachment.*

**Hypothesis** **2a** **(H2a).** *Greenway quality positively contributes to place dependence.*

**Hypothesis** **2b** **(H2b).** *Greenway quality positively contributes to place identity.*

### 2.3. Place Attachment in Relation to Perceived Restoration

Although place attachment can sometimes be negative, it is typically a positive bond. It is believed that place attachment could provide multiple benefits for individuals, such as enhancing individuals’ self-esteem [46], altering attitudes toward an environment [47], improving tourism satisfaction, and improving quality of life [48]. In addition [48,49], studies have highlighted that residents’ place attachment to an environment can make residents favour them, which helps them obtain higher restorative benefits from that environment and further impact their mental wellbeing [50,51,52]. Recent research further revealed that both constructs of place attachment—place dependence and place identity—positively contributed to psychological restoration [53,54]. Using familiar urban park settings, Liu and colleagues found that individuals’ place dependence and place identity play a positive role in enhancing restorative perception in familiar urban park settings [54]. With forest trails, they found that individuals’ place attachment contributes to the increase in visitors’ health [19]. Based on the above statements, this study proposes the following hypothesis:

**Hypothesis** **3** **(H3).** *Place attachment positively contributes to users’ perceived restoration.*

**Hypothesis** **3a** **(H3a).** *Place dependence positively contributes to users’ perceived restoration.*

**Hypothesis** **3b** **(H3b).** *Place identity positively contributes to users’ perceived restoration.*

### 2.4. The Mediating Role of Place Attachment

By providing a space for walking, jogging, and cycling, urban greenways are crucial to urban residents’ health. Research has indicated that higher levels of satisfaction with green space quality were associated with greater place attachment [55,56], and individuals with strong place attachment exhibit more positive emotions and adaptability [57,58] and higher psychological restoration [46,53]. This indicates that place attachment may serve a mediating role between environment quality and perceived restoration. Zhang et al. examined the mediating role of place attachment between neighbourhood green spaces and different features. The results indicated that higher quality neighbourhood green spaces allowed individuals to reap greater psychological health benefits from place attachment. Their study with forest trails further indicated that place attachment plays a partial intermediary role in the influence of recreation involvement on wellbeing [19]. Therefore, based on the above studies, this study proposes the following hypothesis:

**Hypothesis** **4** **(H4).** *Place attachment mediates the relationship between greenway quality and perceived restoration.*

**Hypothesis** **4a** **(H4a).** *Place dependence mediates the relationship between greenway quality of trails and users perceived restoration.*

**Hypothesis** **4b** **(H4b).** *Place identity mediates the relationship between greenway quality of trails and users perceived restoration.*

## 3. Method

### 3.1. Study Area

**Figure 1:**
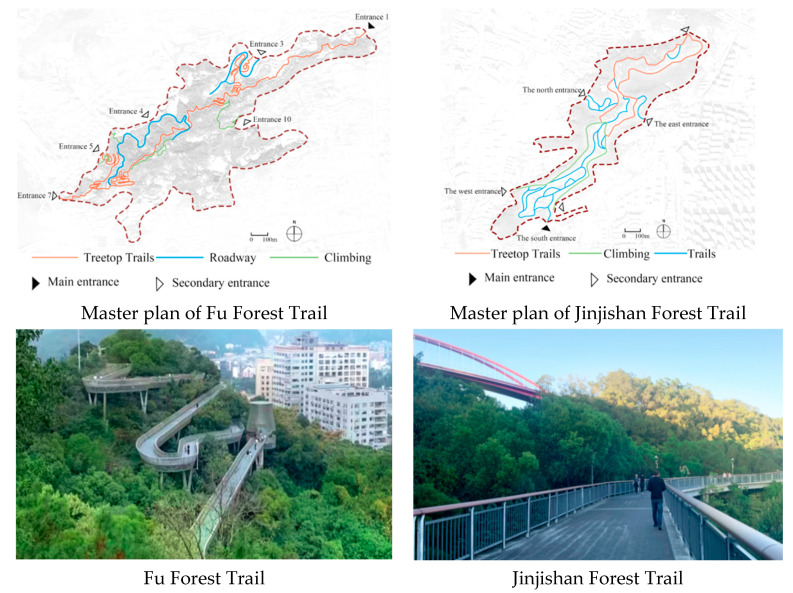
Representative photos of the Fu Forest Trail and Jinjishan Forest Trail.

The survey was conducted in Fuzhou city. Fuzhou is one of the greenest cities in China, and sometimes referred to as Rongcheng or ‘banyan city’, for its numerous banyan trees that line its streets. As a mountainous city, there are several hills in urban area of Fuzhou. To make good use of urban mountains and their associated forest resources, Fuzhou built several treetop trails (called forest walkway trails) [8]. The treetop trails weave through forested areas and provide public access to the green hinterlands. Due to the large number of visitors and compliments for their high quality, the Fu Forest Trail and the Jinjishan Forest Trail (Figure 1) were the most popular of the trails and were selected as case studies by this study. The Fu Forest Trail is the first elevated steel pedestrian walkway of its kind. It spans over 6.3 km from end to end, threading through the full breadth of Jinniushan and covers about 19 km along its winding path [59]. The Jinjishan Forest Trail is located in Jinjishan Park and is the longest sky-view walkway in downtown Fuzhou [60].

### 3.2. Questionnaire Design

The questionnaire consisted of items on demographic information, treetop trails quality, place attachment, and perceived restoration. The demographic information section recorded the participants’ gender, age, and education level. The next section assessed treetop trails quality using a revised perceived quality of treetop trail scale comprising four dimensions perception of trail facilities (PTF), perception of elevated feeling (PEF), perceptions of trail landscape (PTL), perception of trail design (PTD), and twenty-three related indicators [61]. This scale was developed based on previous studies on greenways [62,63] and was modified by the present study according to the characteristics of treetop trails. The Cronbach’s α values (range from 0.88 to 0.98) in the present study indicated that the measurement was reliable. The place attachment section referenced the Place Attachment Scale developed by Williams et al. [36,64], which was divided into two dimensions: place dependence and place identity. The scale has been widely used in studies of places, including national parks, scenic spots, and tourist resorts [52]. The final part assessed the restorative benefits of treetop trails using the Perceived Restorativeness Scale (PRS). PRS was developed by Hartig et al. and was revised by Huang according to the Chinese context. The revised PRS has been extensively used in previous studies and is verified to have good reliability [23,31]. The revised PRS includes four components (including being away, extent, fascination, and compatibility), with 18 indicators [34]. Except for the demographic part, a 7-point Likert scale was used for the assessment in the present study.

### 3.3. Data Collection

Pilot questionnaires were distributed in October 2020 at Fu Forest Trail and Jinjishan Forest Trail for the pre-survey. At the end of the experiment, 53 valid questionnaires were obtained. The data were loaded into SPSS 26 statistical software (IBM Crop., Armonk, NY, USA) for reliability analysis, and the Cronbach’s alpha for the total scale was 0.969, indicating good reliability. The subscales also obtained satisfactory Cronbach’s alphas, with high reliability. Based on the results, the original questionnaire was retained without adjusting the items, and was officially released. In November 2020, a formal questionnaire survey was conducted among the visitors of the treetop trails in Fuzhou city through randomised distribution of on-site paper questionnaires. The investigation period covered sunny days on weekends and weekdays, and the average number of visitors on weekends was 1.2 times that on weekdays. During this period, the COVID-19 epidemic in Fuzhou was relatively stable, with no increase in local cases in the past six months. School was conducted normally. Regarding the prevention and control of the epidemic, the Fuzhou government only required strict control of people from overseas, and local people could move freely. We first observed potential respondents and randomly approached approximately 550 people who we believed to be locals and who had just finished or were taking a walk. Only 493 people agreed to participate in the survey and were asked to complete the questionnaire with instructions. After signing an informed consent, each respondent filled in a paper questionnaire. An average of 40 questionnaires were collected per day and a total of 493 questionnaires were collected, of which 412 were valid, with a questionnaire recovery rate of about 83.5%, which met the reference standard for the sample size for structural equation modelling (SEM) studies [65].

### 3.4. Data Analysis

We performed confirmatory factor analysis (CFA) and SEM to examine the hypotheses. To determine whether the factors and latent variables within the proposed hypothesis were related, CFA assessed the correlation between the two variables. Following Fornell and Larcker’s suggestion [66], we selected composite reliability (CR) and average variance extracted (AVE) as indicators. The CR value represents the internal consistency of the construct indicators, which was suggested to be greater than 0.6. The latent variables’ explanatory power of the measured variables is represented by the AVE value. Hair et al. indicated that the AVE value should be greater than 0.25 [67]. After obtaining the CFA results, we used SEM to test each hypothesis. This study’s goodness-of-fit values included the root mean square error of approximation (RMSEA), the comparative fit index (CFI), the parsimonious CFI (PCFI), the normed fit index (NFI), the Tucker—Lewis index (TLI), and the normed chi-square ratio (χ^2^/df). To ensure that sample size and model complexity did not influence the RMSEA, it must be smaller than 0.08. To explain the model’s simplicity, the PCFI must be greater than 0.05. CFI and NFI demonstrate fitness between the model and null hypothesis and require values greater than 0.095 and 0.09, respectively. Finally, the mediating variables’ effects were analysed according to the path coefficient. Bootstrapping was conducted to examine the statistical significance of the modified model’s indirect effects, total effects, and mediated effects.

## 4. Results

### 4.1. Demographics

Table 1 displays the study participants’ demographic information, which was compared with data provided by Fuzhou. The age of participants ranged between 16 and 66 years, and most participants were aged 18–30. Participants aged 18 and below participated with parental consent. Participants were mostly males (52%), and most were educated to college diplomas (33%), followed by high school diplomas (24%). In general, the characteristics of the participants were similar to those obtained from the Fuzhou data. The participants included a large number of young adults and smaller numbers of older adults and children. This phenomenon is in line with the characteristics of young adults who are active, energetic, and have a large activity radius, as well as the characteristics of the smaller activity radius of older adults [68].

### 4.2. Hypothesis Model

Table 2 shows the questionnaire items, reliabilities, and mean and standard deviation values. The collected questionnaire data were loaded into SPSS 26 and AMOS 21 statistical software for reliability analysis. The alpha coefficient for the total scale was 0.953, and the alpha coefficients for each subscale were all above 0.90, indicating that the observed variables for each latent variable were well designed and that the questionnaire was highly reliable. Bartlett’s test of sphericity and the Kolmogorov–Smirnov test were conducted. The results showed that the *p*-value < 0.001 by Bartlett’s test of sphericity, and the KMO value of the total scale was 0.933. The KMO values for each subscale were greater than 0.70. Therefore, the sample data were well suited for factor analysis.

Before verifying the hypothesis model, we used CFA to identify the relationship between the factors in the hypothesis and the related latent variables. As shown in Table 3, the CFA model met the standards, and the AVE values of all measured variables were greater than 0.5, indicating high convergent validity of each measured variable. Analysis of the correlation matrix between the factors showed that the square root of the AVE of all observed variables was greater than the correlation coefficient between the variables, indicating high discriminant validity of the dimensions. The reliability and validity tests indicated that all questions in the questionnaire had high discriminant validity. Therefore, SEM was used for further verification. The null model’s goodness-of-fit results in this study were as follows: RMSEA = 0.051; PCFI = 0. 883; CFI = 0. 968; TLI = 0.965; NFI = 0.939; and χ^2^/df = 2.046. All the indexes met the value reported by Mulaik et al. [69] these values indicated that the SEM model was acceptable.

**Figure 2:**
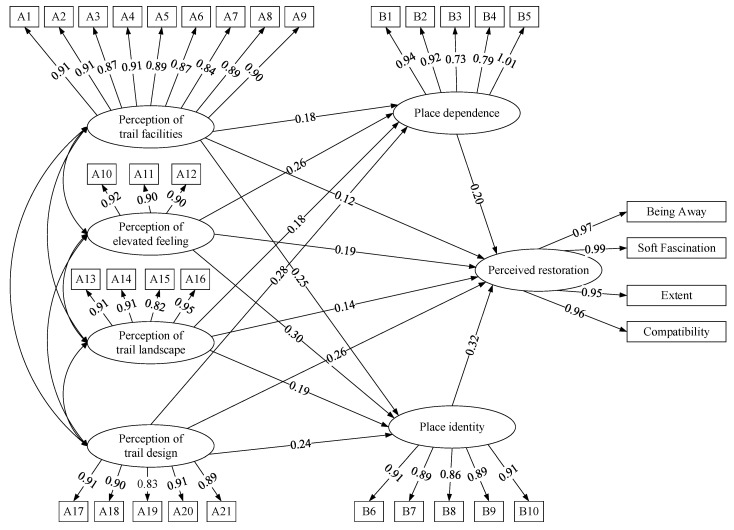
Standard estimates of the Structural Equation Model Path Analysis.

Figure 2 displays the SEM results, where the magnitude of the standardised path coefficients shows the relationship between the individual measurement variables and the level of influence of each measured indicator. The validity of the regression paths in each measurement model can be judged based on the CR value or *p*-value in the table. The path coefficient is significant when CR > 1.96 or *p* < 0.05. As shown in Table 4, the perceived environmental quality of trails (including F1 perception of trail facilities, perception of elevated feeling, perception of trail landscape, and perception of trail design) exerted a significantly positive influence on PR (*p* < 0.001). Among them, PTL had the largest standardised path coefficient, indicating that the perception of trail design had the most significant impact on place identity, followed by PEF, PTL, and PTF. The four components of the quality of the trails exerted significantly positive influences on place identity and dependence. Among them, the standardised coefficients of F4 to F5 and F6 were the largest, indicating that the perception of trail design had the most significant impact on place dependence and place identity. Place attachment exerted a significantly positive influence on users’ perceived restoration (*p* < 0.001). Among them, place identity had the greatest effect on users’ perceived restoration, followed by place dependence. Therefore, **H1, H2a, H2b, H3a**, and **H3b** were supported.

As shown in Table 5, the test for mediation effects showed that place attachment (including place dependence and place identity) exerted a mediating effect on the relationship between quality of trails and users’ perceived restoration, supporting **H4**.

## 5. Discussion

This study investigated relationships among greenway quality, place attachment, and users’ perceived restoration. The results revealed that greenway quality has a positive effect on place attachment and users’ perceived restoration. Place attachment also has a positive effect on users’ perceived restoration with regard to its mediating effect on this relationship.

### 5.1. Quality of Treetop Trails on Users’ Perceived Restoration

One of our aims was to explore users’ sociodemographic characteristics in relation to perceived restoration. The result indicated that the quality of treetop trails had a partial positive influence on users’ perceived restoration, which is consistent with previous studies [25]. In addition, perception of trail design played the most important role in enhancing perceived restoration, which indicated that visitors attach higher importance to the design. Moreover, the users’ sense of safety, comfort, and novelty while walking on the elevated trail may influence their perceived restoration. As unique treetop trails, Fu and Jinjishan forest trails provide public access to the forest areas using an elevated steel walkway system. This also provides the opportunity for the city dwellers to overlook the cityscape. Therefore, there is no doubt that the creative design of the trails simulated a stronger restorative perception.

This is unique to treetop trails compared with normal urban greenways and has a positive impact on users’ perceived restoration.

### 5.2. Quality of Treetop Trails on Place Attachment

Previous studies have established that quality of environments contributed to place dependence [45]. Not only did we validate that this type of relationship applied to the present treetop trails, but we also expanded our knowledge on the particular role of perceived trail design. Perceptions of trail design have the strongest impact on place dependence. This further indicated that users attach great importance to the design of the trail. Therefore, well-designed trails may improve the tendency of users to use the trails and decrease their willingness to use other green spaces. Lewicka’s review [41] demonstrated that individuals would have a stronger place identity with high quality places. In the present study, the perceived environmental quality of the treetop trails had a partially positive influence on place identity, which supported their conclusion. In addition, perception of the elevated trails had the strongest impact on place identity. This may account for the elevated steel pedestrian trails providing an elevated feeling, and they were regarded as the first of their kind in China and the city card of Fuzhou city. Therefore, our study also supported their conclusion that the quality of greenways contributes positively to place attachment [9].

### 5.3. Place Attachment on Users’ Perceived Restoration and Its Mediating Role

Our analysis showed that place attachment had a positive effect on users’ perceived restoration. The path coefficient of the effect of place identity on users’ perceived restoration was higher, at 0.32, indicating that place identity had a greater impact on users’ perceived restoration. The path coefficient of place dependence on users’ perceived restoration was 0.20, indicating that place dependence also had a significant positive effect on users’ perceived restoration. Previous studies indicated that place attachment had a significant positive effect on users’ perceived restoration [70,71]. However, few scholars have conducted comparative analyses on the magnitude of the effects of place dependence and place identity on users’ perceived restoration. Our study shows that place identity plays a more significant role in users’ perceived restoration. Place identity involved users’ attachment to the trail, special meanings attributed to the trail, and how the experience on the trail enriched users’ understanding of themselves, which was more directly and closely related to subjective feelings regarding perceived restoration. In addition, both place dependence and place identity had a partial mediation effect on perceived environmental quality of treetop trails on users’ perceived restoration, which is in line with previous studies [32].

### 5.4. Practical Implications

The present study generated insightful information on how the quality of forest trails contributed to place attachment and perceived restoration. These findings could be used in design processes to provide the public with highly restorative elevated trails. Both the quality of trails and place attachment play positive role in perceived restoration and place attachment plays a mediating role between the quality of trails and perceived restoration. Therefore, designers may need to place a premium on how to make the public build strong attachments to certain environments. In addition, as the quality of trails was assessed from the perception of trail facilities, perception of elevated trails, perception of trail landscape and perception of trail design, designers may focus on the facilities they provide, the elevated attributes, landscape views, and other associated attributes.

### 5.5. Limitations and Further Research

Although this study explored the impact of the perceived environmental quality of treetop trails on users’ perceived restoration through an empirical study, no distinction was made between long-term residents and short-term visitors because of the limited time and scope of the study. Further investigations on the attachment to the environment and its psychological impact on residents and visitors in the same location by classifying users according to the duration of time spent in the local area would be valuable. In his ontology work, Breivik suggests that humans are always connected in a deep way with a ‘world’ of sport, which includes individual sports, encounter sports, team sports, and nature sports. Among them, nature sports refer to the participants’ interaction with nature as the main purpose of the sport. This implied that nature sports may play an important role in determining participants’ place attachment [72]. However, we did not take this into account in the present study. In addition, poor mental health is associated with many negative emotions. We mentioned only a few and did not deal with them all. Further research on the relationship between nature sport and place attachment and other aspects of human mental health should be conducted. Finally, we used subjective measurements for users’ perceived restoration status through questionnaires and the analysis of the questionnaire content. In future, objective experiments such as heart rate and brain wave measurements could be conducted to provide a more comprehensive analysis of the changes in users’ perceived restoration and to further explain the influence mechanisms.

## 6. Conclusions

Development of optimized urban green spaces can only become increasingly important over time as more and more countries become more urbanized. This paper not only demonstrates how place attachment contributes to sense of restoration for visitors using treetop trails but also provides clear guidance on the factors that are most important to consider when planning and developing this particular type of urban greenway. This study indicated that the quality of treetop trails promotes users’ place attachment and perceived restoration. Previous studies have paid more attention to the relationship between activities on the trail and people’s physical health, place attachment, and wellbeing. However, we focused on the quality of treetop trails as the independent variable and obtained the influence mechanisms of that perception on users’ perceived restoration. The perceived environmental quality had a significant positive influence on users’ perceived restoration. Among the environmental quality perceptions, perception of trail design had the greatest influence on users’ perceived restoration, followed by the perceptions of elevated trails, trail landscapes, and trail facilities. Both place dependence and place identity had a significant positive effect on users’ perceived restoration. The path coefficient of place identity on users’ perceived restoration was greater than that of place dependence. Place dependence and place identity had a significant mediation effect on perceived environmental quality of treetop trails on users’ perceived restoration. The results of this study provide guidelines for the construction of future treetop trails.

## Figures and Tables

**Table 1:** Demographic information of study sample.

Variables	Title	Frequency	Surveyed (%)	Fuzhou (%)
Gender	Male	215	52	51.28
	Female	197	48	48.72
Age	18 and below	48	12	NA
	18–30	199	48	NA
	31–40	106	26	NA
	41–60	49	12	NA
	60 and above	10	2	16.76
Education level	Junior high school or below	30	7	NA
	High school	97	24	
	Junior college	43	10	
	College	135	33	
	Master degree or above	107	26	

Note: The population composition of Fuzhou: 17.08% aged 0–14; 66.16% aged 15–59; and 16.76% aged over 60. The number of people with various educational levels per 100,000 people in the city is 18,589 in college; 17,644 in high school; 30,512 in junior high school; and 24,474 in primary school.

**Table 2:** Items, reliabilities, and mean and standard deviation.

Scale Items	Contexts	Means (SD)	Factor Loadings
Perception of trail facilities	A1 Integrity of supporting facilities	4.159 (1.783)	0.911
	A2 Availability of barrier-free facilities	4.007 (1.784)	0.906
	A3 Reasonableness of lighting equipment	4.332 (1.743)	0.867
	A4 Friendliness of rest facilities	4.785 (1.678)	0.911
	A5 Reasonableness of garbage bin placement	3.885 (1.599)	0.889
	A6 Reasonableness of structure distribution	3.788 (1.505)	0.870
	A7 Maintenance of supporting facilities	3.988 (1.726)	0.835
	A8 Completeness of science information distribution facilities	4.527 (1.633)	0.890
	A9 Reasonableness of public toilet distribution	3.734 (1.675)	0.898
Perception of elevated feeling	A10 Safety of overhead passage	4.002 (1.572)	0.917
	A11 Comfort of overhead passage	3.837 (1.585)	0.896
	A12 Novelty of overhead traffic	3.824 (1.742)	0.901
Perception of trail landscape	A13 View of the skyline	3.910 (1.570)	0.909
	A14 Ecology of the surrounding environment	3.778 (1.575)	0.907
	A15 Richness of plants	3.798 (1.662)	0.823
	A16 Viewing of water features	4.185 (1.605)	0.948
Perception of trail design	A17 Comfort of overall colour	3.885 (1.643)	0.911
	A18 Level of adaptation to the terrain	3.732 (1.652)	0.903
	A19 Sense of texture of the materials selected	4.024 (1.773)	0.834
	A20 Level of integration with the environment	4.463 (1.683)	0.908
	A21 Sense of form of the exterior design	3.700 (1.694)	0.894
Place dependence	B1 This is the best place for me to relax and unwind	4.034 (1.554)	0.935
	B2 I am more satisfied with my activities here than on other trails	3.851 (1.488)	0.918
	B3 There is no other trail in my mind that compares to this one	4.017 (1.632)	0.733
	B4 I wouldn’t want to do the activities I do here anywhere else	4.529 (1.607)	0.792
	B5 Although I can perform activities on other trails, this one is more suitable for me	3.646 (1.460)	1.009
Place identity	B6 I feel nostalgic for this trail	3.968 (1.590)	0.910
	B7 I identify emotionally with the trail environment	3.812 (1.593)	0.888
	B8 This trail has special meaning for me	4.178 (1.684)	0.862
	B9 My experience in this trail enriches my understanding of myself	4.649 (1.678)	0.889
	B10 I feel like this trail has become a part of my life	3.807 (1.738)	0.905
Perceived restoration	Being Away	4.051 (1.602)	0.966
	C1 I have an out-of-this-world experience	3.968 (1.757)	0.903
	C2 I get a break from the routine of everyday life	3.778 (1.652)	0.888
	C3 This is where I can rest completely	4.159 (1.801)	0.868
	C4 This place helps me to relax	4.539 (1.713)	0.894
	C5 This place makes me feel free from the constraints of work and daily life	3.812 (1.768)	0.906
	Soft Fascination	4.048 (1.551)	0.986
	C6 The surrounding scenery is coherent	4.151 (1.737)	0.939
	C7 I feel curious about the unseen views in the landscape	4.163 (1.674)	0.941
	C8 This place creates beautiful associations in my mind	3.983 (1.624)	0.900
	C9 The elements of the landscape are well-matched	3.893 (1.588)	0.891
	Extent	4.236 (1.562)	0.950
	C10 This place has attractive qualities	4.156 (1.781)	0.863
	C11 There could be more to explore and discover	4.685 (1.548)	0.891
	C12 The environment is charming	3.876 (1.716)	0.899
	C13 I want to spend more time watching	4.227 (1.653)	0.937
	Compatibility	4.172 (1.465)	0.956
	C14 I can do the activities I like	4.188 (1.363)	0.941
	C15 I can quickly adapt to such scenarios	4.007 (1.585)	0.852
	C16 I feel like I belong here	3.854 (1.550)	0.859
	C17 I can find ways to enjoy myself	4.195 (1.661)	0.851
	C18 The environment facilitates activities that I enjoy	4.615 (1.620)	0.877

**Table 3:** Confirmatory factor analysis.

Factors	Cronbach’s α	Average Variance Extracted	Composite Reliability
Perception of trail facilities	0.935	0.786	0.971
Perception of elevated feeling	0.930	0.819	0.931
Perception of trail landscape	0.952	0.806	0.943
Perception of trail design	0.881	0.661	0.908
Place dependence	0.950	0.776	0.945
Place identity	0.950	0.794	0.951
Perceived restoration	0.981	0.930	0.982

**Table 4:** Tests for mediation effects.

Hypothesis	Direction	*p*	Conclusion
**H1a**	PTF→PR	***	Supported
**H1b**	PEF→PR	***	Supported
**H1c**	PTL→PR	***	Supported
**H1d**	PTD→PR	***	Supported
**H2a1**	PTF→PD	***	Supported
**H2a2**	PEF→PD	***	Supported
**H2a3**	PTL→PD	***	Supported
**H2a4**	PTD→PD	***	Supported
**H2b1**	PTF→PI	***	Supported
**H2b2**	PEF→PI	***	Supported
**H2b3**	PTL→PI	***	Supported
**H2b4**	PTD→PI	***	Supported
**H3a**	PD→PR	***	Supported
**H3b**	PI→PR	***	Supported

Note: *** *p* < 0.001.

**Table 5:** Tests for mediation effects (extended).

Direction	*p*	Direct Effect	Indirect Effect	Total Effect
PTF→PR	***	0.116	0.119	0.235
PEF→PR	***	0.190	0.150	0.341
PTL→PR	***	0.136	0.097	0.233
PTD→PR	***	0.256	0.136	0.392
PTF→PD	***	0.183	0.000	0.183
PEF→PD	***	0.256	0.000	0.257
PTL→PD	***	0.176	0.000	0.176
PTD→PD	***	0.282	0.000	0.282
PTF→PI	***	0.253	0.000	0.253
PEF→PI	***	0.303	0.000	0.304
PTL→PI	***	0.190	0.000	0.190
PTD→PI	***	0.244	0.000	0.244
PD→PR	***	0.204	0.000	0.204
PI→PR	***	0.323	0.000	0.323

Note: *** *p* < 0.001.

## Data Availability

We do not provide public access to the dataset due to protection of the privacy of the participants. Regarding the details of the data, please contact the corresponding author.
